# Clinical Application of Ultraviolet C Inactivation of Severe Acute Respiratory Syndrome Coronavirus 2 in Contaminated Hospital Environments

**DOI:** 10.3390/v13122367

**Published:** 2021-11-26

**Authors:** Wen-Lin Su, Chih-Pei Lin, Hui-Ching Huang, Yao-Kuang Wu, Mei-Chen Yang, Sheg-Kang Chiu, Ming-Yieh Peng, Ming-Chin Chan, You-Chen Chao

**Affiliations:** 1 Division of Pulmonary and Critical Care Medicine, Department of Internal Medicine, Taipei Tzu Chi Hospital, Buddhist Tzu Chi Medical Foundation, New Taipei 23142, Taiwan; dr2639@tzuchi.com.tw (Y.-K.W.); mimimai3461@gmail.com (M.-C.Y.); 2 School of Medicine, Tzu Chi University, Hualien 970, Taiwan; cplincplin8888@gmail.com (C.-P.L.); csk33kimo@hotmail.com (S.-K.C.); chaoycmd@tzuchi.com.tw (Y.-C.C.); 3 Department of Pathology and Laboratory Medicine, Taipei Tzu Chi Hospital, Buddhist Tzu Chi Medical Foundation, New Taipei 23142, Taiwan; jeda377@gmail.com; 4 Division of Infectious Disease, Department of Internal Medicine, Taipei Tzu Chi Hospital, Buddhist Tzu Chi Medical Foundation, New Taipei 23142, Taiwan; mingyieh88@gmail.com; 5 Infection Control Center, Taipei Tzu Chi Hospital, Buddhist Tzu Chi Medical Foundation, New Taipei 23142, Taiwan; jmj621115@gmail.com; 6 Division of Gastroenterology, Department of Internal Medicine, Taipei Tzu Chi Hospital, Buddhist Tzu Chi Medical Foundation, New Taipei 23142, Taiwan

**Keywords:** SARS-CoV-2, COVID-19, environmental sampling, RT-PCR, 254 nm Ultraviolet C

## Abstract

To overcome the ongoing coronavirus disease 2019 (COVID-19) pandemic, transmission routes, such as healthcare worker infection, must be effectively prevented. Ultraviolet C (UVC) (254 nm) has recently been demonstrated to prevent environmental contamination by infected patients; however, studies on its application in contaminated hospital settings are limited. Herein, we explored the clinical application of UVC and determined its optimal dose. Environmental samples (*n* = 267) collected in 2021 were analyzed by a reverse transcription-polymerase chain reaction and subjected to UVC irradiation for different durations (minutes). We found that washbasins had a high contamination rate (45.5%). SARS-CoV-2 was inactivated after 15 min (estimated dose: 126 mJ/cm^2^) of UVC irradiation, and the contamination decreased from 41.7% before irradiation to 16.7%, 8.3%, and 0% after 5, 10, and 15 min of irradiation, respectively (*p* = 0.005). However, SARS-CoV-2 was still detected in washbasins after irradiation for 20 min but not after 30 min (252 mJ/cm^2^). Thus, 15 min of 254-nm UVC irradiation was effective in cleaning plastic, steel, and wood surfaces in the isolation ward. For silicon items, such as washbasins, 30 min was suggested; however, further studies using hospital environmental samples are needed to confirm the effective UVC inactivation of SARS-CoV-2.

## 1. Introduction

Since December 2019, when the coronavirus disease 2019 (COVID-19) was first reported in Wuhan, the severe acute respiratory syndrome coronavirus 2 (SARS-CoV-2) has continued to spread globally, regardless of the season [1]. Therefore, all probable transmission routes, which are mainly via household contacts [2,3] and among healthcare workers (HCWs) [4,5], should be blocked to prevent person-to-person transmission. Regarding HCW infections, the risk factors are exposure, non-use of personal protection equipment, workplace setting, and profession [5]. Environmental service workers in hospitals should observe the proper use of personal protection equipment and hand hygiene and note the influence of profession and knowledge on the contamination of patients’ living areas in the wards to lower their risk of getting infected.

In an isolation ward, SARS-CoV-2 remains on surfaces after shedding, which increases the risk of transmission to HCWs [6]. In particular, SARS-CoV-2 is stable and detectable for up to 72 h on plastic, 48 h on stainless steel, 24 h on cardboard, and 4 h on copper [7]. In another study, SARS-CoV-2 was undetectable on glass and banknotes after four days and on stainless steel and plastic after seven days at room temperature (22 °C) and relative humidity of approximately 65% [8].

In a general hospital setting with temperatures ranging from 20 to 28 °C, and relative humidity ranging from 50 to 80%, these materials are commonly found. Thus, hospital environments provide suitable conditions for SARS-CoV-2 survival [9].

Standard disinfection methods such as bleach were effective in a viral study, and environmental service workers were still in danger if they cleaned the room and treated fomites but did not take the wearing of personal protective equipment seriously [8]. However, Ultraviolet C (UVC) irradiation before bleach cleaning may reduce the infection risk of cleaning service workers. Ultraviolet radiation is effective against viruses, including single-stranded RNA (ssRNA) viruses [10]. SARS-CoV-2, an ssRNA virus [11], can be inactivated after UVC irradiation of viral solutions [12]. However, there are no clinical data collected from hospital SARS-CoV-2 contaminated environments before and after UVC irradiation.

This study aimed to determine the optimal UVC dose required to inactivate SARS-CoV-2 in a clinical hospital setting.

## 2. Materials and Methods

### 2.1. Sample Collection

The first COVID-19 outbreak in Taiwan occurred between 14 May 2021 and 15 August 2021, with 451 COVID-19 patients hospitalized, 112 person-time admissions in isolation intensive care units, and 422 person-time admissions in ordinary isolation wards. The SARS-CoV-2 reverse transcription-polymerase chain reaction (RT-PCR) cycle threshold values (CtVs) were obtained using the nasal swab specimens of these patients at Taipei Tzu Chi Hospital. The study was approved by the Institutional Review Board of the Taipei Tzu Chi Hospital and Buddhist Tzu Chi Medical Foundation (approval number: 10-X-066; date of approval: 16 August 2021) and was conducted according to the guidelines of the amended Declaration of Helsinki. Informed consent was waived by the institutional review board because patients’ data were anonymized.

### 2.2. Sampling Locations

COVID-19 patients were admitted to either the ordinary ward or the intensive care unit (ICU) with standard negative-pressure isolation rooms or a single room with slight negative pressure. In standard negative-pressure rooms, the negative-pressure differences ranged from −14 to −15 Pa between the ward and buffer areas and −8 to −9 Pa between the clean corridor and buffer area. Air exchange was performed 12 times per hour. The ward temperature ranged from 23.4–24.1 °C, and relative humidity ranged from 58.2–59.3%. The ICU had temperatures ranging from 24.8–28.9 °C, and relative humidity of 55.4–62.5%.

A heat and moisture exchange filter (VH-3110 FHME, Great Group Medical Co., Ltd., Changhua, Taiwan) was installed for intubated patients to prevent the virus from entering the mechanical ventilator via the tubing loop. To prevent aerosol outbreaks during sputum suction, a closed suction device (T20005 PAHSCO, Pacific Hospital Supply Co., Ltd., Miaoli, Taiwan) was also connected. For non-ventilator patients, surgical masks were worn after low- or high-flow nasal cannula therapy.

Environmental cleaning was performed regularly every 3 days in the ward and buffer areas using sodium hypochlorite (5–6%) solutions (bleach) diluted at 1:100 (*v*/*v*) for floor application and 1:10 for other surfaces.

The decrease in the viral load in the upper respiratory tract of a patient with a CtV < 25 may span a longer duration [13]. In our preliminary study, of the 72 environmental samples collected from the ward of a patient with nasal swabs SARS-CoV-2 RT-PCR CtVs > 25 tested absolutely negative for viral contamination. Therefore, samples were not collected in this case. However, samples from the buffer area (i.e., the ICU and the ward) were collected as control groups. Environmental samples were collected every week from the ward where a COVID-19 patient with a nasal swab SARS-CoV-2 CtV < 25 was admitted for at least 3 days.

Regular environmental sampling aimed to ensure regular bleach cleaning and further immediate cleaning if SARS-CoV-2 was detected on the surfaces of the ward or working place. Environmental samples were collected from 8–10 sites before routine cleaning using sterile throat swabs (LIBO Specimen Collection and Transport Swabs, LIBO Medical Products Inc., New Taipei, Taiwan) and transported in a viral transport medium (CMP^®^, New Taipei, Taiwan). To increase the positive sampling rate, samples were collected from three different locations on each surface of the equipment. Based on the shape of the surface at the environmental facilities, a total of 15 cm^2^ area was selected in each facility and divided into three 5 cm^2^ locations. The environmental samplings were randomly sampled using sterile throat swabs from three different locations. To evaluate the dose–effect relationship, environmental samples were recollected after UVC irradiation.

### 2.3. SARS-CoV-2 RT-PCR Analysis

All samples were analyzed for SARS-CoV-2 using RT-PCR at a biosafety level 2 (BSL-2) laboratory with a built-in negative pressure room for immediate RT-PCR testing and the UVC dosing effects were compared [14]. Specific RT-PCR targeting the RNA-dependent RNA polymerase and the *E* and *N* genes were used to detect the presence of SARS-CoV-2 according to the WHO guideline [15]. Samples were stored at 4 °C and analyzed within 2 days if they were obtained routinely. Nucleic acids were extracted using a LabTurbo Viral DNA/RNA Mini Kit on a LabTurbo^TM^ 48 Compact System, Taipei, Taiwan. Briefly, 0.5 mL preservation solution were added to the swab samples, and 25 μL proteinase K and 0.5 mL VXL buffer were placed into a new sample tube. The solution was mixed with a micropipette 3–5 times. The positive extraction control (PEC) and negative extraction control (NEC) were also prepared. The nucleic acid extraction process included decomposing the virus particles in the sample, binding the virus nucleic acid to the tube column membrane, cleaning the silicon membrane, and recovering the virus nucleic acid from the membrane.

For the *E*, *N*, and *RdRP* genes, a 25 µL reaction was performed with 6 µL template RNA and 19 µL PCR master mix, and RNA was detected with a LabTurbo^TM^ AIO COVID-19 RNA testing kit (Cat. No. Acov11240, Taipei, Taiwan) on a LabTurbo^TM^ 48 AIO system (Lab Turbo Biotech Co., Ltd., Taipei, Taiwan). Thermal cycling was performed at 55 °C for 10 min for reverse transcription, followed by heat activation at 95 °C for 1 min and 45 cycles of amplification at 95 °C for 10 s (denaturation) and 60 °C for 15 s (annealing/extension). The human *RNAse P* gene was used as the extraction control and the secondary negative control [16]. For nasal swab specimens, a positive finding was defined as the lowest value of the *E*, *N*, or *RdRP* gene. For the environmental samples, any detection of the *E*, *N*, or *RdRP* gene CtV was concluded as a positive finding of environmental contamination. During the COVID-19 pandemic, to quickly complete a large number of SARS-CoV-2 RT-PCR tests, the *RdRP* gene was only tested when any *E* gene or *N* gene in the environmental sample was positive. The number of cycles required for the fluorescent signal to cross the threshold during RT-PCR was determined as CtVs. In general, lower CtVs were associated with higher viral loads.

Contamination rate (%) was calculated as follows: number of positive findings/total number of environmental samplings × 100%.

### 2.4. UVC Irradiation

Irradiation was performed at 254 nm (UVC) by a hyperlight disinfection robot (Mediland Co., Ltd., Taoyuan, Taiwan). We calculated a dose of 42 mJ/cm^2^ after 5 min of UVC irradiation of a single room with an estimated width of 3 m. The dose-related cleaning effects were assessed after irradiation for 5, 10, 15, 20, and 30 min with estimated energy doses of 42, 84, 126, 168, and 252 mJ/cm^2^, respectively. Mediland Co., Ltd. (Taoyuan, Taiwan) calculated the estimated irradiation dose (mJ/cm^2^) after 5–30 min of irradiation with 3 m width. The irradiation dose was measured using UV energy meters.

To set up the 254-nm UVC irradiation standard procedure for environmental cleaning, a series of studies were conducted to test its dose-related effect on SARS-CoV-2 contaminated environments. Twelve locations in the isolation ward were selected for environmental sampling before cleaning. 

### 2.5. Statistical Analysis

Continuous data are presented as the mean ± standard error. Categorical data are expressed as frequencies and percentages. The demographic and environmental characteristics of the ward and ICU groups were compared using Student’s *t*-test. Analysis of variance was used for comparisons of data for the ward, ICU, and buffer areas. The chi-square test was used to compare the categorical data for the ward and ICU groups based on different UVC irradiation doses. The linear trends of the dose-related effects of UVC irradiation on SARS-CoV-2 were also tested using the chi-square test. Data were analyzed using SPSS (version 24.0; IBM Corp., Armonk, NY, USA), and statistical significance was set at *p* < 0.05.

## 3. Results

**Figure 1:**
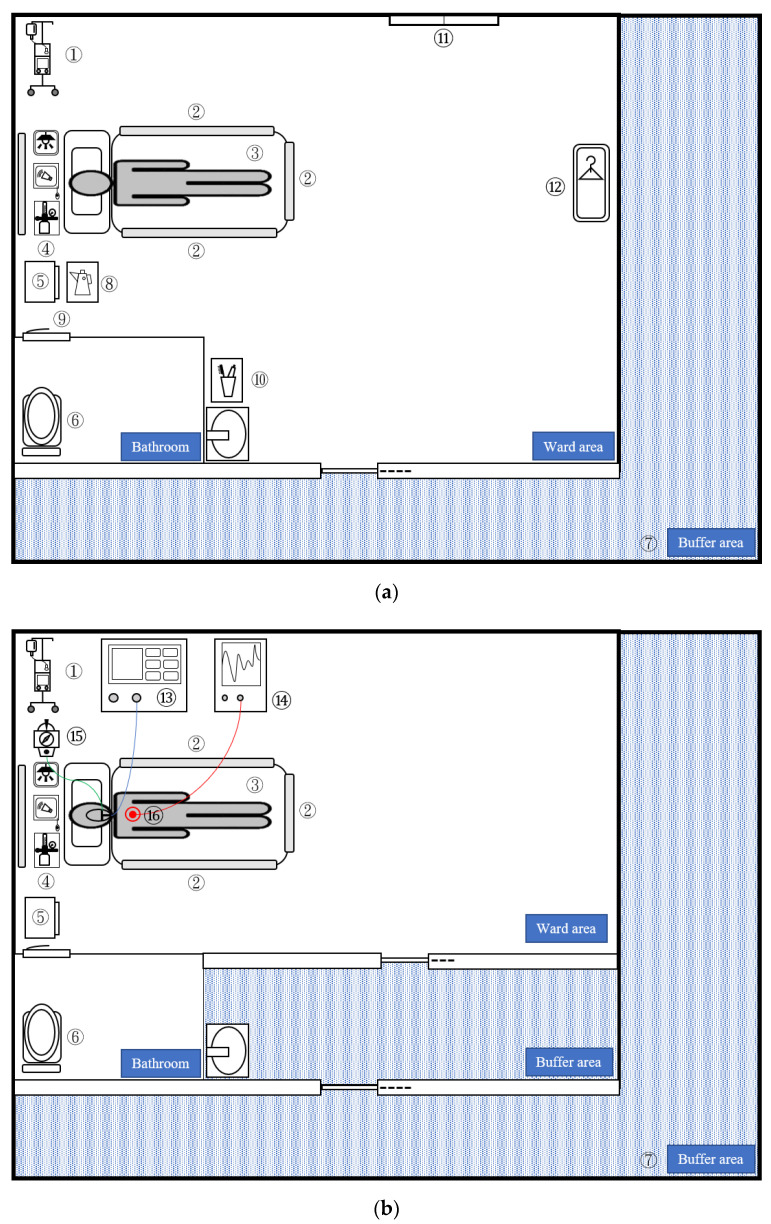
Illustration of COVID-19 isolation wards (**a**) and ICUs (**b**) indicating the sampling areas. The contamination rate of each area is as follows: Common area: ① IV stand and IV pump (rate = 0/11, 0%); ② bed rail (rate = 4/52, 7.7%); ③ mattress (rate = 0/11, 0%); ④ wall equipment: call bell, oxygen flowmeter, light switch (rate = 0/17, 0%); ⑤ bedside table (rate = 3/25, 12%) ⑥ toilet: toilet seat and flush button (rate = 2/11, 18.2%); ⑦ buffer area (rate = 0/50, 0%); Exclusive ward: ⑧ kettle (rate = 2/12, 16.7%); ⑨ door handle (rate = 0/12, 0%); ⑩ washbasin including toothbrush and mouth cup (rate = 5/11, 45.5%) ⑪ windowsill (rate = 1/5, 20%); ⑫ wardrobe (rate = 0/5, 0%); Exclusive ICU: ⑬ ventilator (rate = 0/11, 0%); ⑭ monitor (rate = 0/11, 0%); ⑮ suction switch (rate = 1/11, 9.1%); and ⑯ EKG lead (rate = 1/12, 8.3%). COVID-19, coronavirus disease-19; ICU: intensive care unit.

A total of 267 routine environmental samples were collected from the ward, the ICU, and the buffer area during the study period (Table 1). There were 16 SARS-CoV-2–positive samples and 86 negative samples in the ward (contamination rate 15.7%); 3 and 112 in the ICU (contamination rate 15.7%); and 0 and 50 in the buffer area (contamination rate 0%). The washbasin (45.5%), windowsill (20%), and kettle (16.7%) in the isolation ward had the highest SARS-CoV-2 environmental contamination (Figure 1a). The contamination rates were 18.2%, 12%, and 7.7% for the toilet, bedside table, and bed rails, respectively. The contamination rate of the suction switch was 9.1%, and that of the electrocardiogram lead was 8.3% in the ICU (Figure 1b). The nasal swab CtVs of patients in the ward were significantly lower than those in the ICU (mean ± SE: 20.7 ± 0.5 vs. 22.9 ± 0.6; *p* < 0.001). The overall contamination rates were 15.7%, 2.6%, and 0% in the ward, ICU, and buffer areas, respectively (*p* < 0.001 in Table 1). Similar trends were obtained for the *E*, *N*, and *RdRP* genes, with the highest contamination rate found in the ward vs. ICU (8.8% vs. 2.6%, 15.7% vs. 1.7%, and 6.9% vs. 2.6%, respectively) and 0% in the buffer area. The *E* gene CtV of the ward was significantly lower than that of the ICU (31.8 ± 0.7 vs. 36.4 ± 2.1, *p* = 0.02). The human *RNAse P* gene positivity rates in the ward, ICU, and buffer areas were 43.1%, 23.5%, and 16%, respectively; the differences were significant (*p* < 0.001), indicating that ambulatory patients in the ward may have higher environmental human cell detection rates. However, the CtV of the human *RNAse P* gene did not significantly change (*p* = 0.430).

In the subgroup analysis (Table 1 and Figure 1), the contamination rates of the “common area” were the same for the ward and ICU. The ward had higher *N* gene (14%, *p* = 0.011) and human *RNAse P* gene (35.1%, *p* = 0.011) contamination rates than the ICU (1.4% and 15.7%, respectively). In the other subgroup analysis, the exclusive ward and ICU were compared. We found that the ward had higher contamination (17.8%, *p* = 0.045) and *N* gene contamination rates (17.8%, *p* = 0.03) than the ICU (4.4% and 2.2%, respectively).

The patient’s nasal swab SARS-CoV-2 RT-PCR CtV was 13 (Table 2). Before irradiation, five (41.7%) locations were positively contaminated. After exposure to UVC radiation for 5 min, 10 min, and 15 min, the contamination rate decreased to 25%, 8.3%, and 0%, respectively (*p* = 0.005). The human *RNAse P* gene positivity rate also decreased from 58.3% (control) to 25%, 16.7%, and 16.7% after UVC irradiation for 5 min, 10 min, and 15 min, respectively, (*p* = 0.025). Human cells were also destroyed by UVC irradiation; therefore, patients should leave the isolation ward before the UVC cleaning procedures. 

We also determined the optimal UVC irradiation period for SARS-CoV-2 environmental disinfection. Repeated studies on the dose-effect of UVC irradiation were conducted for 15–30 min on the second day of regular patient activity (Table S1). SARS-CoV-2 was inactivated after 15 min of UVC irradiation; however, one area in the washbasin tested positive for the presence of the *N* gene (8.3) after 20 min of irradiation. At 30 min of UVC irradiation, all areas tested negative for the presence of SARS-CoV-2 genes (Table S1). The human *RNAse P* gene positivity rate decreased from 41.7% to 8.3%, 16.7%, and 8.3% after UVC irradiation for 15 min, 20 min, and 30 min, respectively (*p* = 0.118). On the fourth day of regular patient activity, the washbasin was divided into four areas for sampling (Table S2). Before irradiation, two of these areas (50%) tested positive for the *E* and *N* genes. Bleach cleaning was performed on areas C and D. After 15 min of UVC irradiation, no SARS-CoV-2 genes were detected.

## 4. Discussion

Surface contamination in SARS-CoV-2 isolation wards may have occurred without environmental cleaning, especially in those places where patients were admitted within 3 days with a nasal swab SARS-CoV-2 RT-PCR CtVs < 25. The isolation ward had a higher contamination rate than the ICU, especially for the washbasins. UVC irradiation at 254 nm for at least 15 min was effective for the inactivation of SARS-CoV-2. However, for silicon-containing surfaces, such as washbasin and other surfaces with a slightly high relative humidity, UVC irradiation for 30 min is suggested.

As the isolation ward had a higher contamination rate, it was selected for further tests. Desimmie et al. reported that the SARS-CoV-2 CtVs of nasal swabs of patients admitted to the isolation ward between the incubation period and symptomatic onset day were low [17]. Seven days after symptom onset, patients became critically ill; therefore, we suggest that the CtV was higher in patients admitted to the ICU than in those admitted to the isolation ward. As shown in Table 1, the CtVs of samples from the ward were significantly lower than those from the ICU. This is supported by the higher infection risk in the isolation ward than in the ICU. 

In our study, plastic and silicone items, such as toothbrushes, mouth cups, kettles, suction buttons, washbasins, bedside tables, and toilets, had the highest contamination rates. SARS-CoV-2 was also detected on stainless steel surfaces (bed rails and EKG lead). Meanwhile, wood surfaces (wardrobe, windowsill) were less contaminated. These findings are attributed to the tendency of SARS-CoV-2 to stay longer on non-porous surfaces than porous surfaces [18]. The presence of sunlight is also a factor that contains UVC natural disinfection, and temperature and humidity conditions affect the longevity of SARS-CoV-2 (some windowsill and wardrobe) [9,19,20].

The UVC irradiation doses varied in different studies. For ssRNA viruses, the UVC irradiation dose required for 90% viral reduction ranged from 1.32–3.2 mJ/cm^2^ [10]. Twice the dose of radiation exposure was predicted for 99% viral reduction. Another study reported that the complete inactivation of SARS-CoV-2 can be achieved after 16.9 mJ/cm^2^ of UVC irradiation [12]. Shimoda et al. evaluated the effect of UVC wavelength on SARS-CoV-2 inactivation [21] and reported that for 3-log reductions in viral titers, irradiation doses of 4.5 mJ/cm^2^, 4.4 mJ/cm^2^, and 8.2 mJ/cm^2^ at 265 nm, 254 nm, and 280 nm UVC were required. The 254-nm and 265-nm UVC dose were lower than 280-nm for the inactivation of SARS-CoV-2. Ruetalo et al. investigated the dose-dependent effect of 254 nm UVC irradiation on the inactivation of SARS-CoV-2 in a six-well plate [22]. High titer surface dried SARS-CoV-2 (3 to 5 × 10^6^ IU/mL) and was totally inactivated by 254 nm UVC at 16 mJ/cm^2^. Furthermore, a dose of 1500 mJ/cm^2^ was suggested for 10 µL of SARS-CoV-2 viral stock (8 × 10^7^ TCID_50_/mL) based a simulation of SARS-CoV-2 contamination of N95 respirators [23]. However, this study focused on personal protective equipment and not on the devices or equipment found in contaminated isolation rooms. Our study is the first study to investigate the effect of UVC irradiation on contaminated surfaces of hospital environments; 254 nm UVC irradiation at 15–30 min (126–252 mJ/cm^2^) was suggested for the total inactivation of SARS-CoV-2. Compared with other SARS-CoV-2 studies, our study showed that the dose required for 254 nm UVC disinfection of environmental surfaces was lower than that of N95 respirators (1500 mJ/cm^2^) but higher than that of basic research (16 mJ/cm^2^). 

Human *RNAse P* gene is not always detected in the environment except if patients or healthcare workers touch the environmental facilities. Therefore, the human *RNAse P* gene cannot be used as an internal control in environmental samplings. However, the human *RNAse P* gene can implicate SARS-CoV-2 environmental contamination and may be related to human transmission. In our study results (Table 1 and Table 2), each SARS-CoV-2 RT-PCR positive result also showed a positive result for the human *RNAse P* gene at the same time. The buffer area was negative for SARS-CoV-2, and a 16% positivity rate for the human *RNAse P* gene was observed (Table 1), indicating that human cells remained in the buffer areas and that routine environmental cleaning is required despite negative findings for SARS-CoV-2 RNA. 

UVC radiation exhibits a bactericidal effect on glass, plastic, and steel surfaces and is less effective on those of Teflon and silicon [24]. In our study, washbasins and toilets had higher SARS-CoV-2 contamination (Figure 1 and Table S2). The washbasin required 30 min of radiation to inactivate SARS-CoV-2 (Table 2). Washbasins and toilets contain silicon materials and are usually located in rooms with relatively low temperatures without sunlight. Regarding washbasins, water splashing frequently occurs when patients gargle. The water-containing surfaces may have higher humidity; the relative humidity of the washbasin was 70–73% higher than that of the other surfaces. The high humidity may also decrease the UVC inactivation effect, and it may be necessary to increase the radiation dose [10]. We showed that the washbasin required UVC irradiation for 30 min to ensure the total eradication of the virus. 

Kitagawa et al. reported that 1–3 mJ/cm^2^ of 222 nm UV irradiation reduced the SARS-CoV-2 viral load by 88.5–99.7%, which was detected as the 50% tissue culture infectious dose (TCID_50_) [25]. In their study, the viable virus cultures seem more sensitive and suitable than the RT-PCR in detecting the dose-effect of UVC irradiation on viral suspensions at a relatively high concentration of 5 × 10^6^ TCID_50_/mL. However, our clinical trace virus environmental study did not elucidate the relationship between CtVs and viable SARS-CoV-2 contamination, as we could not perform this because of the unavailability of a BSL level III laboratory. Therefore, future studies should perform environmental sampling on SARS-CoV-2 cultures or in vitro plaque assays to clarify this relationship.

## 5. Conclusions

We demonstrated that UVC irradiation at 254 nm, at an estimated dose of 126 mJ/cm^2^ is effective for environmental cleaning of plastic, steel, and wood surfaces in a clinical isolation ward for SARS-CoV-2 inactivation. For water containers and silicon items, such as washbasins, with low temperature and slightly high relative humidity, we suggest higher doses of 252 mJ/cm^2^ UVC irradiation; however, further studies are required to confirm the optimal irradiation dose of UVC.

## Figures and Tables

**Table 1:** Detection of SARS-CoV-2 and gene contamination in different hospital environments (*N* = 267).

SARS-CoV-2 RT-PCR	Ward (*n* = 102)	ICU (*n* = 115)	Buffer Area (*n* = 50)	*p*
Patient CtV, mean ± SE	20.7 ± 0.5	22.9 ± 0.6	NA	0.004 *
Overall contamination rate, *n* (%)	16 (15.7)	3 (2.6)	0	<0.001 *
human *RNAse P* gene ^a^, *n* (%)	44 (43.1)	27 (23.5)	8 (16.0)	<0.001 *
CtV, mean ± SE	33.3 ± 0.4	32.4 ± 0.8	33.5 ± 1.2	0.430
*E* gene, *n* (%)	9 (8.8)	3 (2.6)	0	0.021 *
CtV, mean ± SE	31.8 ± 0.7	36.4 ± 2.1	NA	0.02 *
*N* gene, *n* (%)	16 (15.7)	2 (1.7)	0	<0.001 *
CtV, mean ± SE	31.7 ± 0.6	31.9 ± 0.7	NA	0.930
*RdRP* gene, *n* (%)	7 (6.9)	3 (2.6)	0	0.078
CtV, mean ± SE	31.0 ± 0.4	32.3 ± 0.4	NA	0.128
Contamination Rate in Different Areas				
Common area, *n* (%)	8/57 (14.0)	1/70 (1.4)	NA	0.011 *
human *RNAse P* gene, *n* (%)	20/57 (35.1)	11/70 (15.7)	NA	0.011 *
CtV, mean ± SE	33.5 ± 0.7	32.9 ± 1.3	NA	0.688
*E* gene, *n* (%)	4/57 (7.0)	1/70 (1.4)	NA	0.173
CtV, mean ± SE	32.4 ± 0.9	33.2	NA	0.716
*N* gene, *n* (%)	8/57 (14)	1/70 (1.4)	NA	0.011 *
CtV, mean ± SE	31.5 ± 0.7	31.1	NA	0.861
*RdRP* gene, *n* (%)	3/57 (5.3)	1/70 (1.4)	NA	0.218
CtV, mean ± SE	31.0 ± 0.4	32.6	NA	0.192
Exclusive ward or ICU, *n* (%)	8/45 (17.8)	2/45 (4.4)	NA	0.045 *
human *RNAse P* gene, *n* (%)	24/45 (53.3)	16/45 (35.6)	NA	0.09
CtV, mean ± SE	33.2 ± 0.5	32.1 ± 1.0	NA	0.301
*E* gene, *n* (%)	5/45 (11.1)	2/45 (4.4)	NA	0.434
CtV, mean ± SE	31.4 ± 1.1	38.0 ± 2.4	NA	0.03 *
*N* gene, *n* (%)	8/45 (17.8)	1/45 (2.2)	NA	0.03 *
CtV, mean ± SE	31.8 ± 1.0	32.6	NA	0.818
*RdRP* gene, *n* (%)	4 (8.9)	2 (4.4)	NA	0.398
CtV, mean ± SE	31.0 ± 0.8	32.1 ± 0.7	NA	0.349

CtV, cycle threshold value; SE, standard error; NA, not applicable. The categorical variables are presented as *n* (%), *n*: presented as the number of positive results of environmental sampling, (%): presented as the percentage of positive rate. The continuous variables are presented as mean ± SE; ^a^ the human *RNAse P* gene was detected as a nucleic acid extraction procedural control and secondary negative control. (* *p* < 0.05).

**Table 2:** Dosage effect of 254-nm UVC radiation at various irradiation periods.

*n* = 12	Control	Irradiation for 5 min	Irradiation for 10 min	Irradiation for 15 min	*p*
Positivity rate, *n* (%)	5 (42)	3 (25)	1 (8)	0 (0)	0.045 ^a^
human *RNAse P*, *n* (%)	7 (58)	3 (25)	2 (17)	2 (17)	0.077 ^b^
CtV, mean ± SE	32.5 ± 0.9	32.0 ± 0.2	32.4 ± 1.2	33.7 ± 1.5	0.837
*E* gene, *n* (%)	3 (25)	0 (0)	1 (8)	0 (0)	0.088 ^c^
CtV, mean ± SE	30.4 ± 1.1	NA	34.8	NA	0.188
*N* gene, *n* (%)	5 (42)	3 (25)	1 (8)	0 (0)	0.045 ^d^
CtV, mean ± SE	30.2 ± 0.9	33.8 ± 0.8	33.1	NA	0.071
*RdRP* gene, *n* (%)	3 (25)	1 (8)	1 (8)	0 (0)	0.237 ^e^
CtV, mean ± SE	30.3 ± 0.6	31.4	35.3	NA	0.105

Patient’s nasal swabs SARS-CoV-2 RT-PCR CtV was 13. CtV, cycle threshold value; SE, standard error; NA, not applicable. The categorical variables are presented as *n* (%), *n*: presented as the number of positive results of environmental sampling, (%): presented as the percentage of positive rate. The continuous variables are presented as mean ± SE. ^a^ *p* = 0.005 significance for linear trend, dose effect; ^b^ *p* = 0.025 significance for linear trend, dose effect; ^c^ *p* = 0.064; ^d^ *p* = 0.005 significance for linear trend, dose effect; ^e^ *p* = 0.06.

## Data Availability

The data that support the findings of this study are available from Taipei Tzu Chi Hospital, Buddhist Tzu Chi Medical Foundation, but restrictions apply to the availability of these data, which were used under license for the current study, and they are not publicly available. The data are, however, available from the authors upon reasonable request and with permission from Taipei Tzu Chi Hospital, Buddhist Tzu Chi Medical Foundation.

## Supplementary Materials

The following are available online at https://www.mdpi.com/article/10.3390/v13122367/s1, Table S1: The dosage effect of 254 nm UVC radiation on different time; Table S2: The 254 nm irradiation on washbasin.
