# Supplementary material for: Clinical Application of Ultraviolet C Inactivation of Severe Acute Respiratory Syndrome Coronavirus 2 in Contaminated Hospital Environments
Source: Viruses. 2021 Nov 26;13(12):2367. doi: 10.3390/v13122367 (PMC8706350; doi:10.3390/v13122367)
Supplement: Supplementary file 1 [file viruses-13-02367-s001.zip › viruses-1409020-supplementary.pdf]

Supplementary Material

Clinical Application of Ultraviolet C Inactivation of Severe Acute Respiratory Syndrome Coronavirus 2 in Contaminated Hospital Environments

Table S1. The dosage effect of 254 nm UVC radiation on different time.

CtV = 13, *n* = 12	Control	Irradiation for 15 min	Irradiation for 20 min	Irradiation for 30 min	*p*-value
Positive rate, *n* (%)	5 (42)	0 (0)	1 (8)	0 (0)	0.005[a]
human *RNAse P* gene, *n* (%)	5 (42)	1 (8)	2 (17)	1 (8)	0.118[b]
CtV, mean±SE	33.2±1.9	32.2	34.9±1.2	32.8	0.928
E gene, *n* (%)	1 (8)	0 (0)	0 (0)	0 (0)	0.382[c]
CtV, mean±SE	29.5	NA	NA	NA	NA
N gene, *n* (%)	5 (42)	0 (0)	1 (8)	0 (0)	0.005[d]
CtV, mean±SE	32.9±1.4	NA	34.2	NA	0.720
RdRP gene, *n* (%)	2 (17)	0 (0)	0 (0)	0 (0)	0.100[e]
CtV, mean±SE	32.1±3.4	NA	NA	NA	NA

UVC, ultraviolet C; CtV, cycle threshold value; SE, standard error; NA, not applicable. The categorical variables are presented as n (%), *n*: presented as the number of positive results of environmental sampling, (%): presented as the percentage of positive rate. The continuous variables are presented as mean ± SE. a.-$p = 0.001$ significance for Linear trend, dose effect b.-$p = 0.031$ significance for Linear trend, dose effect. c-$p = 0.109$. d.-$p = 0.001$ significance for Linear trend, dose effect. e.-$p = 0.022$ significance for Linear trend, dose effect.

Table S2. The 254 nm irradiation on washbasin.

CtV = 18, *n* = 4	A	B	C	D
Before irradiation	+	–	+	–
human *RNAse P* gene	+	–	+	–
CtV	28.86	NA	28.16	NA
E gene	+	–	+	–
CtV	34.53	NA	32.47	NA
N gene	+	–	+	–
CtV	31.55	NA	29.16	NA
RdRP gene	–	–	–	–
Bleach Tx	No	No	Yes	Yes
UVC 15 min	–	–	–	–
human *RNAse P* gene	–	+	–	–
CtV	NA	35.22	NA	NA
E gene	–	–	–	–
N gene	–	–	–	–
RdRP gene	–	–	–	–
UVC 20 min	–	–	–	–
human *RNAse P* gene	+	–	–	–
CtV	36.1	NA	NA	NA
E gene	–	–	–	–
N gene	–	–	–	–
RdRP gene	–	–	–	–
UVC 30 min	–	–	–	–
human *RNAse P* gene	+	–	–	–
CtV	34.2	NA	NA	NA

E gene	–	–	–	–
N gene	–	–	–	–
RdRP gene	–	–	–	–

CtV, cycle threshold value; UVC, ultraviolet C; NA, not applicable. Bleach cleaning was done on position C and D. +, any SARS-CoV-2 gene was detected; –, none of SARS-CoV-2 gene was detected.
